# Heat-induced transformation of nickel-coated polycrystalline diamond film studied in situ by XPS and NEXAFS

**DOI:** 10.3762/bjnano.16.67

**Published:** 2025-06-12

**Authors:** Olga V Sedelnikova, Yuliya V Fedoseeva, Dmitriy V Gorodetskiy, Yuri N Palyanov, Elena V Shlyakhova, Eugene A Maksimovskiy, Anna A Makarova, Lyubov G Bulusheva, Aleksandr V Okotrub

**Affiliations:** 1 Nikolaev Institute of Inorganic Chemistry, SB RAS, 630090 Novosibirsk, Russia; 2 Sobolev Institute of Geology and Mineralogy, SB RAS, 630090 Novosibirsk, Russia; 3 Physical Chemistry, Institute of Chemistry and Biochemistry, Free University of Berlin, 14195 Berlin, Germany; 4 Helmholtz-Zentrum Berlin für Materialien und Energie, 14109 Berlin, Germany

**Keywords:** graphitization, near-edge X-ray absorption fine structure spectroscopy, nickel coating, polycrystalline diamond film, single-crystal diamond, X-ray photoelectron spectroscopy

## Abstract

Controlling high-temperature graphitization of diamond surfaces is important for many applications, which require the formation of thin conductive electrodes on dielectric substrates. Transition metal catalysts can facilitate the graphitization process, which depends on the diamond face orientation. In the present work, the role of a nickel coating on the electronic structure and chemical state of graphite layers formed on the surface of a polycrystalline diamond (PCD) film with mixed grain orientation was studied. A synthetic single-crystal diamond (SCD) with a polished (110) face was examined for comparison. The samples were coated with a thin nickel film deposited by thermal evaporation. The graphitization of diamond with and without a nickel coating as a result of high-vacuum annealing at a temperature of about 1100 °C was studied in situ using synchrotron-based X-ray photoelectron spectroscopy (XPS) and near-edge X-ray absorption fine structure (NEXAFS) methods. XPS data revealed the formation of a thin graphite-like film with low-ordered atomic structure on the surface of the nickel-coated PCD film. The chemical state of sp^2^-hybridized carbon atoms was found to be insensitive to the face orientation of the diamond micro-sized crystallites; however, the layer defectiveness increased in areas with fine-dispersed crystallites. According to NEXAFS and Raman spectroscopy data, the most ordered atomic structure of graphitic layers was obtained by annealing nickel-coated SCD. The angular dependence of NEXAFS C K-edge spectra of nickel-coated (110) face after annealing discovered the vertical orientation of sp^2^-hybridized carbon layers relative to the diamond surface. The observed behavior suggests that sp^2^ carbon layers were formed on the diamond surface due to its saturation by released carbon atoms as a result of etching by nickel.

## Introduction

Diamond and graphite, both composed entirely of carbon atoms, exhibit vastly different properties due to their distinct atomic structures. Diamond is a wide bandgap semiconductor, which makes it resistant to high voltages and ionizing radiation. In contrast, graphitic materials demonstrate excellent electrical conductivity. This divergence in physical properties has encouraged significant interest in producing hybrid materials which combine these two forms of carbon [1–3]. In particular, such graphene-on-diamond heterostructures have been shown to be attractive for power electronics [4–5], microelectronic devices [6–7], and detectors [7–8].

At room temperature and atmospheric pressure, carbon in sp^3^ hybridization is a metastable material. A significant activation barrier hampers its relaxation into sp^2^ graphitic carbon, and this transformation occurs during vacuum heating in the temperature range of 1500–1800 °C [9]. According to molecular dynamics simulations, graphitization of nonterminated diamond surfaces is initiated at 750 °C. A temperature of about 1500 °C is needed for the formation of extended graphene-like layers, and temperatures higher than 2000 °C are required for the complete conversion of the diamond (111) surface to graphitic layers [10–11]. Thermal stability of diamond crystals depends on the crystallographic orientation of their faces [12–13]. In particular, the (100) face exhibits greater resistance to annealing compared to that of the (111) face [10,13–15], and the (110) face has proven to be the most unstable when exposed to high temperatures [14,16].

The coating of diamond surface with a metal catalyst has been explored to reduce the temperatures required for the initiation of the graphitization process. Nickel [17–24], iron [25–28], copper [29–30], gallium [31], and molybdenum [32] allow the fabrication of graphene-on-diamond heterostructures by annealing. Among those, nickel attracts specific attention since the 1960s [33] because its lattice parameter is close to that of diamond. Single-crystal diamond (SCD) substrates were subjected to nickel-assisted graphitization [17–21]. The transformation of the SCD surface into graphene requires annealing at temperatures above 800 °C [21]. The annealing of nanocrystalline diamond (NCD) films in the presence of a Ni catalyst has been recently explored [22–24]. It was shown that graphitization of Ni-coated NCD films begins at a relatively lower temperature of about 500 °C [23]. Such a significant decrease in the temperature at which graphitization starts compared to that of the Ni-coated SCD is due to the presence of multiple grain boundaries, along which the diffusion of Ni atoms takes place, facilitating the graphitization process [34].

The process occurring at the interface between diamond and Ni nanoparticles was revealed using high-resolution transmission electron microscopy (HRTEM) [19,24]. During annealing, Ni nanoparticles etch the diamond surface, resulting in the formation of a narrow interdiffusion zone. The carbon atoms released from the diamond surface diffuse across the Ni surface. After the Ni particles are saturated with carbon, the excess carbon precipitates to form the sp^2^-hybridized graphitic layers parallel to the Ni surface [19,24]. Alternatively, these atoms could diffuse along the etched diamond surface, saturating the dangling bonds and producing the sp^2^ carbon on the free diamond surface, or diffuse into the Ni bulk, feeding the graphite formation from the side of the catalytic particle [19]. Comparing the morphology of Ni-coated SCDs annealed under similar conditions revealed the anisotropic nature of both the diamond etching [35–36] and the graphitization of the diamond surface [19]. In particular, the (111) face was found to be resistant to etching, producing a thin layer of disordered graphite that was weakly bonded to the underlying diamond surface but strongly attached to the Ni particles. In contrast, the Ni nanoparticles penetrate beneath the (110) and (100) surfaces, creating pits that were partially filled with graphite covalently bonded to the etched diamond surface.

From prior works, it can be seen that the Ni-assisted graphitization of diamond has been studied on either SCDs, which have better properties but high cost, or on more affordable NCD films, whose properties are notably inferior to their monocrystalline counterparts. In this regard, microcrystalline diamond (MCD) films could serve as a more suitable alternative to SCDs. Therefore, the Ni-assisted graphitization of MCD films requires a detailed study. HRTEM has proven very useful for investigating the graphite–diamond interface [19,24]. However, it provides information about local morphology and ordering of diamond surface and graphite layers. X-ray photoelectron spectroscopy (XPS) and near-edge X-ray absorption fine structure (NEXAFS) methods are noncontact and nondestructive methods to investigate the chemical state of the elements on the surface and in the bulk of solids. The signals collected over a large surface area provide overall insight into the surface state. Moreover, the polarization-dependence of NEXAFS spectra provides information about the spatial orientation of π and σ orbitals relative to the photon incidence.

In the present work, we focused on the changes in the surface state of Ni-coated polycrystalline diamond (PCD) films composed of micron-sized grains with (110) and (111) faces during high-vacuum annealing at a temperature of about 1100 °C. To exclude the influence of impurities from the air, the annealed samples were examined in situ using XPS and NEXAFS without contact with the air. XPS and NEXAFS spectroscopy investigations of heat-induced transformation of the surface of bare and Ni-coated PCD films were conducted at the experimental station of the Russian–German Beamline using the BESSY II synchrotron radiation facility. To achieve variable depth sensitivity, XPS spectra were collected at two excitation photon energies, and NEXAFS spectra were recorded using two registration modes. Additionally, angle-resolved NEXAFS spectra of annealed Ni-coated SCD were measured to reveal the orientation of the formed graphitic layers. After synchrotron measurements, the samples were exposed to air and further analyzed using Raman spectroscopy and scanning electron microscopy (SEM). The obtained results revealed detailed information about the morphology of the graphitized layer formed on the PCD film surface during annealing in the presence of nickel. We were also able to determine the texture of these graphitized layers relative to the (110) face of SCD.

## Results and Discussion

### Surface transformation of bare and nickel-coated polycrystalline diamond films under high-vacuum annealing

The PCD film was produced by plasma-enhanced chemical vapor deposition (PE CVD) using acetone (CH_3_)_2_CO, hydrogen, and air as the precursor gases for the plasma [37]. The film consists of crystallites with nonuniform geometry, dimensions, and orientation (Supporting Information File 1, Figure S1a–c). The large diamond micro-sized crystallites, measuring about 100 μm, have a complex cuboctahedron shape with facets that have straight and acute angles. Electron backscatter diffraction (EBSD) analysis detected (110) and (111) crystallographic planes on the surface of the PCD film (Supporting Information File 1, Figure S1b). The mapping did not show regions with the (100) orientation, although cubic faces are visible in the SEM images. The signal from these faces is probably weakened due to the tilt of the crystallites and the rough PCD film. Various growth defects, including pits, cracks, steps, and protrusions are present on the diamond faces. The secondary nucleation of diamond caused the formation of submicron-sized diamond grains and smoothing of the shape of large crystals. Raman spectroscopy revealed high crystalline quality in the PCD film at the micron scale (Supporting Information File 1, Figure S2). Thermal evaporation of nickel and its deposition on the PCD film surface resulted in the formation of a uniform metallic layer with a thickness of about 40 nm (Supporting Information File 1, Figure S1d). The bare PCD film and that with a nickel coating (denoted Ni-PCD) were placed on the same holder and simultaneously annealed in the vacuum chamber of the RGL-PES end-station of BESSY at 1100 °C for 15 min. After annealing, PCD and Ni-PCD films were cooled to room temperature without contact with air and examined in situ using NEXAFS and XPS methods. The changes in the chemical state of the surface of the PCD and Ni-PCD films as a result of annealing were examined.

**Figure 1:**
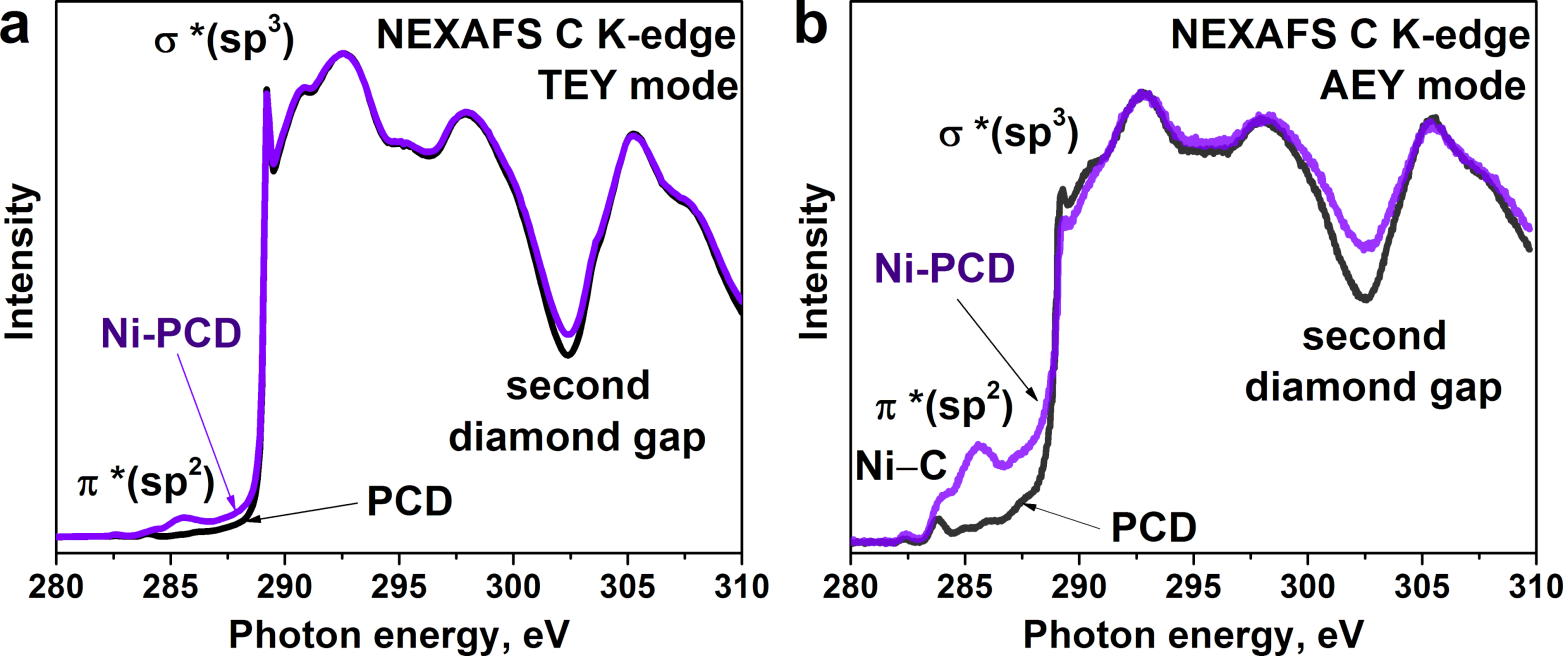
NEXAFS C K-edge spectra of PCD and Ni-PCD films after high-vacuum annealing at 1100 °C, measured in a) TEY mode and b) AEY mode.

The NEXAFS C K-edge spectra were simultaneously recorded in total electron yield (TEY) and Auger electron yield (AEY) modes to probe the volume (10 nm) and the surface (3 nm) of the films, respectively (Figure 1). The C K-edge spectra of the annealed samples show a sharp peak at 289.3 eV assigned to the electron transition from 1s to unoccupied σ* states within the sp^3^-hybridized carbon atoms in the diamond (σ*(sp^3^)) and a wide dip at 302.2 eV corresponding to a second absolute gap in the diamond band structure [38]. In the TEY spectra of both PCD and Ni-PCD films, the aforementioned spectral features are well pronounced, indicating the preservation of the ordered crystalline structure of diamond in the bulk of the film after annealing (Figure 1a). In the AEY spectra of both films, the smoother shape of the σ*(sp^3^) resonance and the shallower dip suggest to the presence of structural disorders on the surface of diamond films (Figure 1b). The amount of these disorders in Ni-PCD is higher than that in PCD. This result confirms previous findings that the metal catalyst induces the formation of disordered carbon on the diamond surface during annealing [21–23]. All spectra also show a weak feature at 285.5 eV, which corresponds to the electron transitions from C 1s to unoccupied π* states in sp^2^-hybridized carbon species (π*(sp^2^)). It was found that 1s‒π*(sp^2^) excitations in the aromatic molecules and graphene also contribute to the spectral region between 286.0 and 288.5 eV, albeit with a low intensity [39–40].

The π*(sp^2^) peak is more intense in the AEY spectra than in the TEY spectra, meaning that the film surface consists of carbon atoms in sp^2^-hybridized state. The π*(sp^2^) peak in the spectra of the annealed Ni-PCD film has a much higher intensity than that in the spectra of the annealed PCD film. Based on this observation, we can conclude that nickel promotes the conversion of the diamond film surface into an sp^2^-carbon coating upon annealing. This result confirms previously reported findings, which demonstrated the catalytic role of nickel in the reconstruction of a diamond surface [17–24]. Based on electron microscopy and Raman spectroscopy data, the authors of those studies claimed that graphite or graphene-like layers are products of the diamond annealing process.

The intensity ratio of π*(sp^2^)- and σ*(sp^2^)-resonances in NEXAFS C K-edge spectra of the annealed samples can be used for qualitative assessment of structural perfection in a graphitic-like material. Graphite and graphene have a high degree of local crystallinity (i.e., high ordering of carbon atoms in the honeycomb network) and their C K-edge spectra contain a narrow and intense π*(sp^2^)-resonance [41–42]. However, the rather low intensity of the π*(sp^2^)-resonance in the spectrum of the annealed Ni-PCD film indicates that annealing of polycrystalline films on average leads to the formation of structurally highly disordered forms of sp^2^ carbon layers. The AEY spectra of the annealed PCD and Ni-PCD films exhibit an additional pronounced feature at about 284.0 eV, which can also be assigned to π*(sp^2^)-resonance and associated with the presence of large aromatic fragments on the surface of both annealed films [43]. Moreover, this peak overlaps with the characteristic C K-edge features of transition metal carbides. Therefore, it can also be attributed to the presence of Ni‒C states in the annealed Ni-PCD film [44].

Survey XPS spectra of the annealed samples showed a strong C 1s line at ≈285 eV and a weak Ni 3p peak at ≈67 eV only for the Ni-PCD film (Supporting Information File 1, Figure S3). Oxygen and other elements were not detected on the surface of the samples. The low surface concentration of nickel (0.1 atom %) could be associated with the heat-induced reorganization of the Ni layer into particles, which can penetrate into the diamond substrate due to the counter-diffusion of carbon and nickel [19]. The immersing of metal particles into diamond was discussed in detail in previous works [27–28].

**Figure 2:**
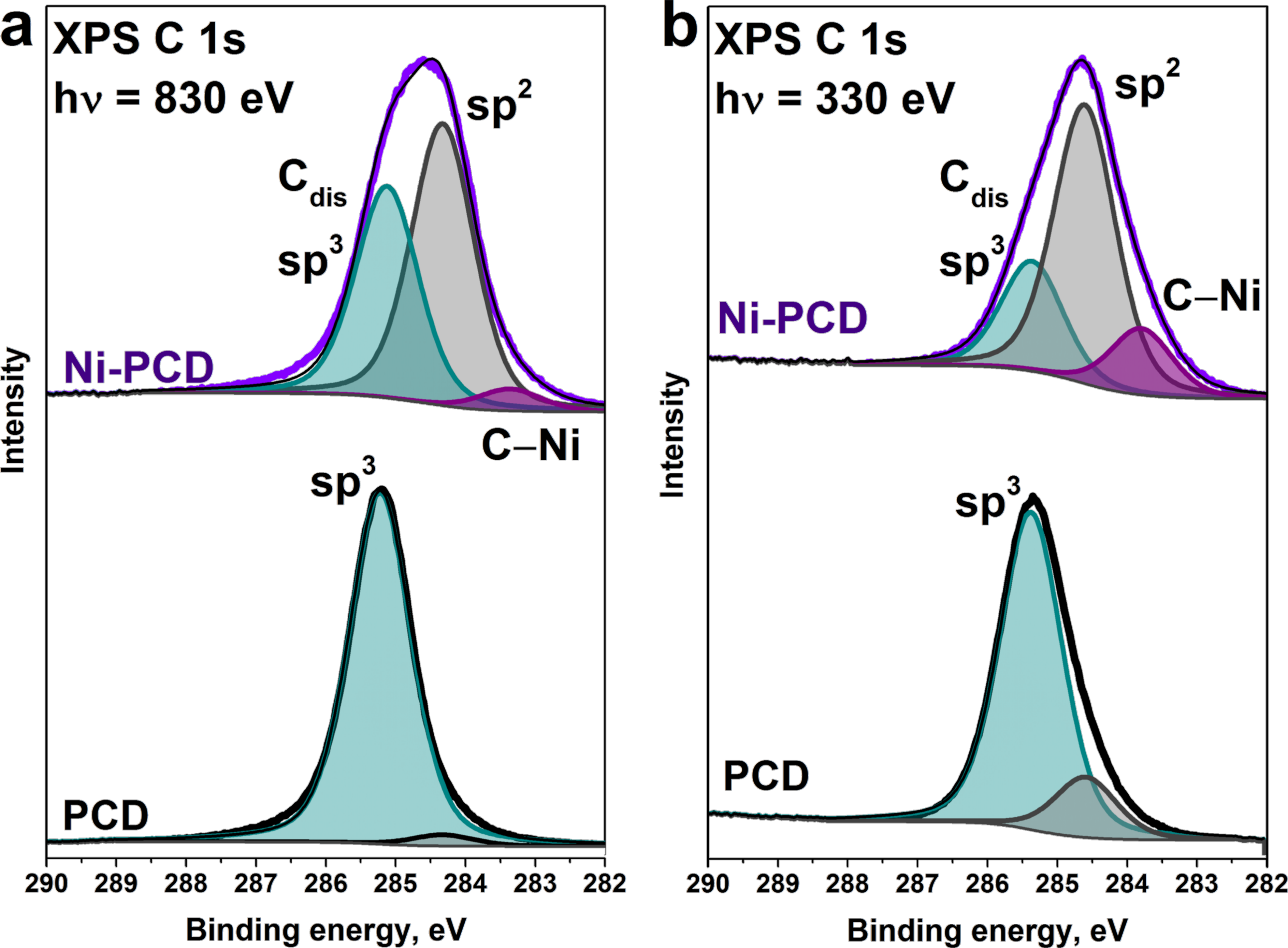
XPS C 1s spectra of PCD and Ni-PCD films after high-vacuum annealing at 1100 °C, measured at excitation photon energies of a) 830 eV and b) 330 eV.

The XPS C 1s spectra were measured upon excitation by photons with energies of 830 and 330 eV to probe different surface depths of the samples after annealing (Figure 2). In these cases, the inelastic mean free path for electrons emitted from the C 1s level in diamond is about 1.0 nm (probing depth of 3 nm) for 830 eV and 0.7 nm (probing depth of 2.1 nm) for 330 eV, respectively [45]. The C 1s spectrum of PCD after high-vacuum annealing was fitted with two components. The dominant peak at 285.2 eV is assigned to sp^3^-hybridized carbon atoms in diamond crystals. Additionally, there is a tiny peak at 284.3 eV assigned to sp^2^-hybridized carbon atoms. The relative area of this sp^2^-peak is 3% in the spectrum measured at 830 eV, and becomes significantly larger (16%) as the photon excitation energy decreases to 330 eV. The rather small amount of sp^2^ carbon in the 2 nm thick surface layer indicates that the temperature and duration of the annealing process were not sufficient to achieve significant graphitization of the PCD surface without a nickel layer. In contrast, an intense sp^2^-carbon component is observed in the C 1s spectra of Ni-PCD, confirming that the diamond surface in the presence of nickel catalyst more readily transforms to sp^2^-hybridized carbon. For excitation at 830 eV, the sp^2^ peak is quite broad (1.1 eV) compared to that in the spectrum of a highly ordered graphite crystal (0.6 eV) [42]. The reason for this is the high density of defects in the carbon layer formed on the Ni-PCD surface during annealing. In the spectrum of the annealed Ni-PCD surface, the peak at 285.2 eV can be assigned to diamond sp^3^-states similar to that in the initial diamond. However, highly disordered sp^3^ carbon states (C_dis_) can also give rise to this peak. According to a recent XPS study of the graphitization process of Ni-coated NCD films, a disordered carbon was found to form on the nickel surface, which then partially transformed into a graphitic phase at higher temperatures [23]. Moreover, the spectra of the annealed Ni-PCD film demonstrate an additional peak at a low binding energy of 283.3 eV corresponding to carbon bonded with nickel (denoted as C‒Ni in Figure 2). The amount of C‒Ni states decreases as the probing depth increases. The XPS data are consistent with the AEY NEXAFS spectrum of the annealed Ni-PCD, confirming that the Ni coating facilitates the transformation of the diamond surface upon heating, resulting in the development of a thin sp² carbon layer over the entire surface of the sample.

**Figure 3:**
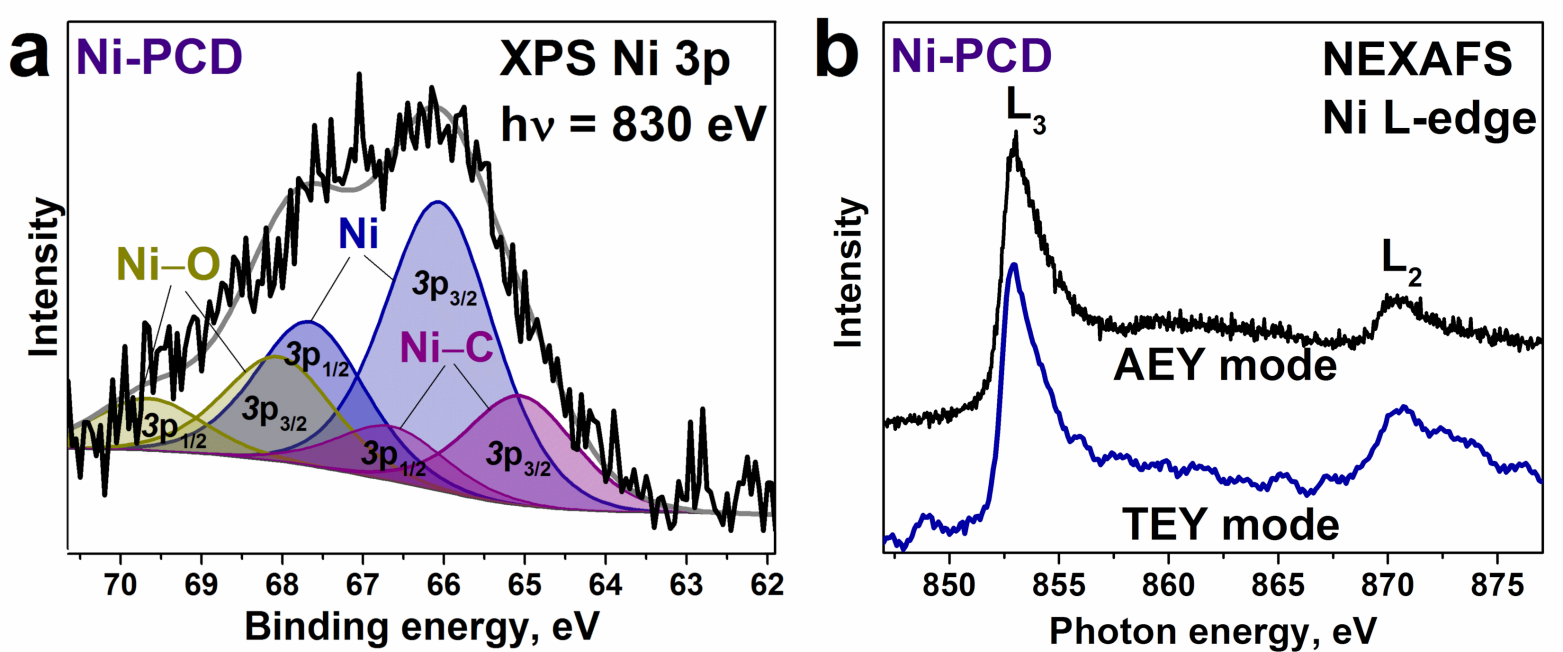
a) XPS Ni 3p spectrum measured at 830 eV and b) NEXAFS Ni L-edge spectra recorded in TEY and AEY modes of Ni-PCD film after high-vacuum annealing at 1100 °C.

The chemical state of nickel in the annealed Ni-PCD film was elucidated using XPS and NEXAFS spectroscopy to probe the local environment of metal atoms on the surface and inside the bulk (Figure 3). The XPS Ni 3p spectrum was fitted by three doublets, related to the spin–orbit splitting into Ni 3p_3/2_ and Ni 3p_1/2_ components, separated by 1.6 eV (Figure 3a). The most intense doublet with the Ni 3p_3/2_ component at 66.1 eV is attributed to metallic nickel [46]. The high-energy doublet with the Ni 3p_3/2_ component at 68.1 eV corresponds to the oxidized states of nickel (Ni–O). The appearance of these states may be due to the interaction of nickel with residual water in the vacuum chamber or with oxygen desorbed from the silicon substrate during annealing [47]. The low-energy doublet with the Ni 3p_3/2_ component at 65.0 eV can be referred to nickel bonded with carbon (Ni–C) [48]. The Ni‒O and Ni‒C doublets contribute no more than 14% to the total spectral area (with a surface content of less than 0.01 atom %). The NEXAFS Ni L-edge spectra measured in TEY and AEY modes show peaks at 852.7 and 870.4 eV, corresponding to L_3_ and L_2_-edges, respectively (Figure 3b). According to their energy positions, the metallic nature of the nickel appears to dominate both in the bulk and on the surface of Ni-PCD after annealing [49].

**Figure 4:**
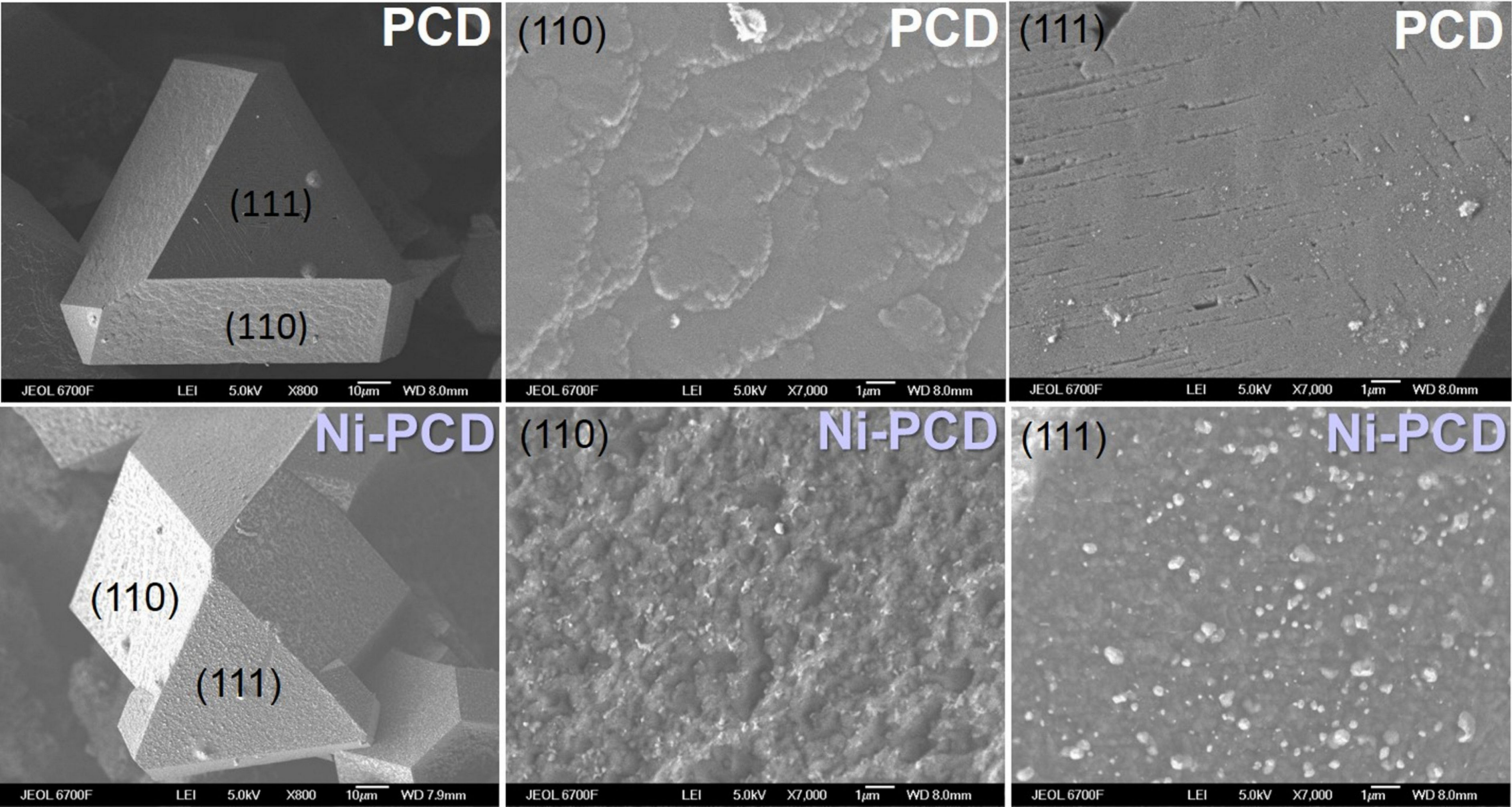
SEM images of crystallites with different faces in annealed PCD and Ni-PCD films.

After completion of synchrotron investigations, the PCD and Ni-PCD films were removed from the vacuum chamber for further SEM and Raman analysis under ambient conditions. Figure 4 shows SEM images of some large crystallites of about 100 μm in size on the surfaces of the annealed PCD and Ni-PCD. These crystallites have well-defined triangular (111) faces and truncated rectangular faces, which could be assigned to either the (110) or (100) planes. Since the (110) orientation of grains strongly dominates, as shown by the EBSD map, the rectangular faces will be referred to as (110). The bare film almost completely preserved its initial morphology after annealing. A close examination revealed that the diamond faces are sufficiently flat and show no signs of thermal degradation. In the annealed Ni-PCD film, the crystallites have a rougher surface. SEM images do not allow for a definite confirmation of whether sp^2^ carbon is present on the surface, even near defective states, partly due to its fine structure.

Comparison of SEM images taken from two different faces of the Ni-coated crystallite reveals that the initially solid nickel layer is rearranged into particles whose shape and distribution depend on the orientation of the diamond face (bright spots in bottom panels in Figure 4). In particular, on the rectangular (110) faces, nickel particles are flatter and more evenly distributed than those on the triangular (111) face. Previous studies showed that the etching of diamond through the reaction with Ni during annealing is an anisotropic process [19,35–36]. In particular, the (100) and (110) faces are etched simply, while the (111) face is flattened during the process. We assume that small nickel nanoparticles formed above the (110) faces are embedded in the diamond, while their agglomerates remain on the (111) faces, appearing as large particles enclosed in carbon shells. Element mapping analysis based on energy-dispersive X-ray (EDX) spectroscopy revealed a uniform distribution of nickel particles in the top layers of all diamond faces after annealing (Supporting Information File 1, Figure S4). The low resolution of the EDX instrument used did not allow the detection of nickel-free areas of diamond surfaces.

**Figure 5:**
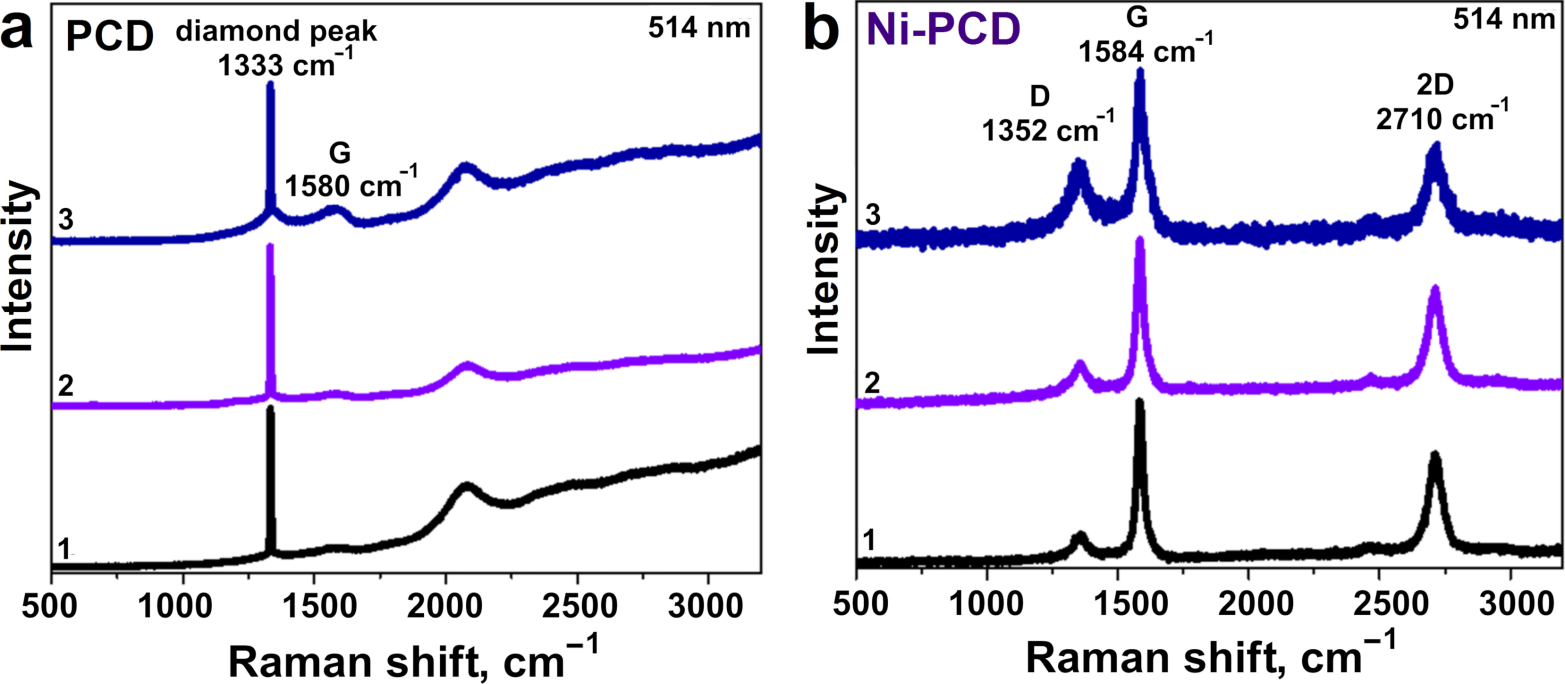
Raman spectra of a) PCD and b) Ni-PCD films after annealing in high vacuum at 1100 °C. The spectra were registered from two different faces of a diamond microcrystal (1 and 2) and from the region between microcrystals (3).

Raman spectroscopy was used to compare the different regions of the annealed PCD and Ni-PCD films (Figure 5 and Supporting Information File 1, Figure S5). The spectra recorded for two different faces of large microcrystallites in the annealed PCD film look similar (Figure 5a, Supporting Information File 1, Figure S5a,b). They demonstrate the main diamond peak at 1333 cm^−1^ corresponding to the first-order scattering of the F_2g_ symmetry. The high intensity and small full width at half maximum (FWHM) of 4 cm^−1^ of this band and the absence of other Raman features indicate the high crystallinity of the sp^3^ lattice and the low concentration of nondiamond phases in the annealed PCD. The spectrum taken from the area between the microcrystallites, in addition to the diamond band, shows a weak Raman signal from the sp^2^-hybridized carbon, namely a broad G band at 1580 cm^−1^ from C=C stretching vibrations. This indicates that in our experimental conditions, the partial graphitization of bare PCD film occurs more actively in the areas with small crystallites enriched with boundaries and defects, while large crystallites retain their diamond structure.

The out-of-focus Raman spectrum of the annealed Ni-PCD film taken from a large area demonstrates intense Raman peaks from both diamond and sp^2^-carbon (Supporting Information File 1, Figure S6). In contrast, the Raman spectra taken from two different faces of large crystallites and from an area with small crystallites in the annealed Ni-PCD film only demonstrate the Raman peaks from sp^2^-carbon and the absence of the diamond peak at 1333 cm^−1^ (Figure 5b, Supporting Information File 1, Figure S5c-e). The probing depth of Raman scattering is estimated to be about 90 nm (Supporting Information File 1, Table S1). This suggests that nondiamond components with a thickness of no less than 90 nm, consisting of sp^2^-carbon and Raman-inactive nickel particles, uniformly cover the faces of large diamond crystallites. In addition to the G band at 1584 cm^−1^, there are two distinct peaks at 1352 and 2710 cm^−1^, corresponding to the D and 2D bands. The D band represents the disordered vibration modes of graphitic hexagonal layers, and the 2D band originates from the second-order double-resonant scattering process. In general, the quality of graphene layers can be evaluated by the ratio of the intensities of the D and G peaks (*I*_D_/*I*_G_). The spectrum recorded from the area containing small diamond crystallites exhibits the highest *I*_D_/*I*_G_ value of 0.43, which is approximately twice as high as the *I*_D_/*I*_G_ value of 0.25 for the faces of the large diamond crystallite. This indicates that the sp^2^-hybridized carbon layers formed during the catalytic graphitization of small crystallites contain more defects compared to those formed on the continuous surface of micro-sized crystallites. The number of graphitic layers in the carbon coating forming the graphitized surface of the annealed Ni-PCD film can be analyzed by the ratio of the intensity of 2D and G peaks (*I*_2D_/*I*_G_) and the FWHM of the 2D peak [50]. A monolayer graphene typically has the *I*_2D_/*I*_G_ values greater than 2 and the FWHM of the 2D peak of ≈30 cm^−1^. In all spectra measured from different areas of the annealed Ni-PCD film, the *I*_2D_/*I*_G_ value is about 0.6 and the FWHM of the 2D peak is ≈90 cm^−1^. This suggests the formation of multilayer graphitic stacks on different faces of the annealed Ni-PCD film.

To summarize this section, diamond microcrystallites are highly resistant to transformation into sp^2^ carbon as a result of vacuum annealing at a temperature of 1100 °C. An exception is smaller diamond crystallites, whose surfaces partially transform into amorphous sp^2^-like carbon. The presence of a nickel layer promotes the conversion of the diamond surface into graphitic-like thin films with high concentration of structural defects. Although the morphology of nickel nanoparticles varies depending on the orientation of the diamond face, we did not observe differences in the chemical state of sp^2^ carbon located in the same regions. This suggests that the structure of the graphitic-like coating formed during Ni-assisted graphitization depends to a small extent on the crystallographic orientation of the diamond surface, and is mainly determined by the annealing temperature. On the other hand, the defectiveness of the sp^2^ layers is influenced by the crystallite size and the presence of intrinsic structural defects in the diamond.

### Orientation of graphitic layers on the (110) face of single-crystal diamond after annealing at 1150 °C

In order to eliminate the role of nickel in the reconstruction of the (110) diamond surface and the orientation of sp^2^ carbon layers, we coated the (110) face of SCD with a thin nickel film of the same thickness as that on the PCD. Next, we annealed it and conducted angle-dependent TEY NEXAFS measurements at the C K-edge.

**Figure 6:**
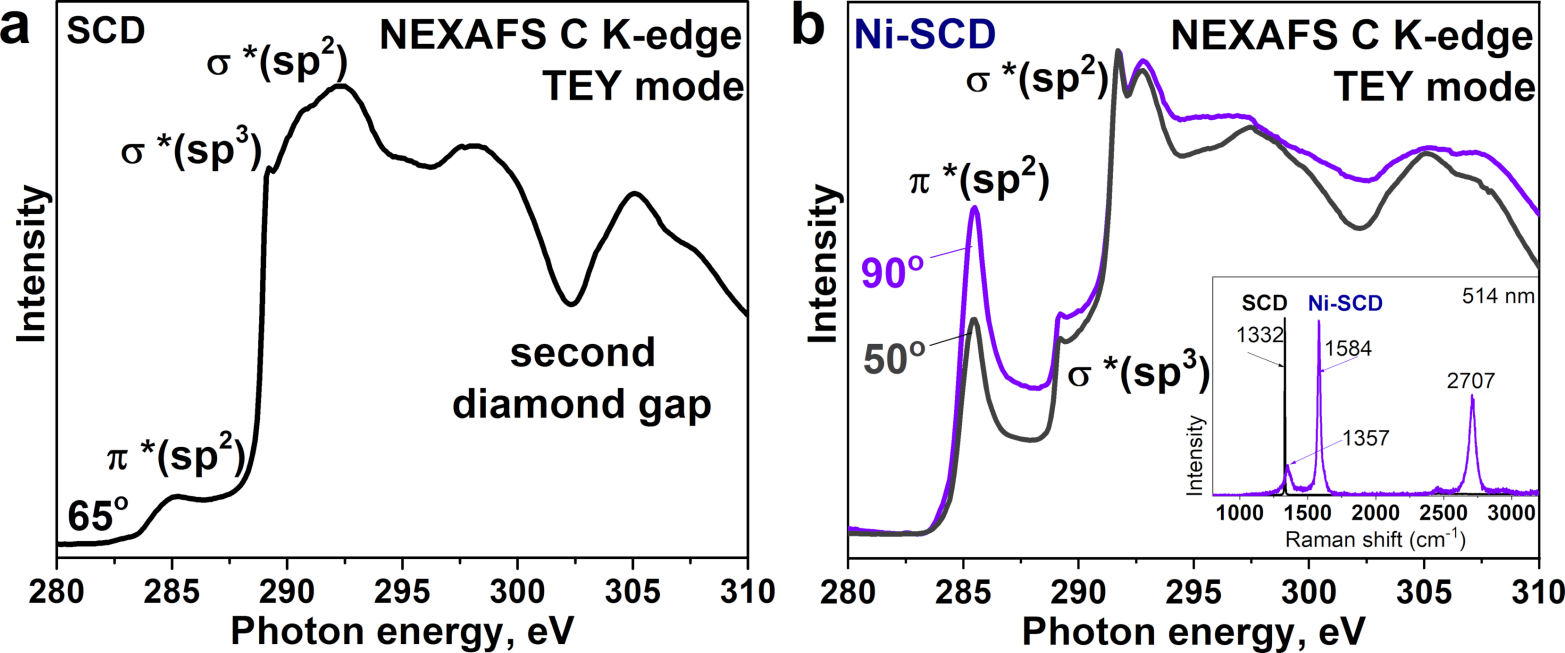
NEXAFS C K-edge spectra measured in TEY mode for a) bare and b) Ni-coated (110) face of SCD after high-vacuum annealing at 1150 °C. The angle of incidence of the synchrotron radiation was a) 65° for SCD and b) 50 and 90° for Ni-SCD. The inset in panel (b) shows Raman spectra of annealed SCD and Ni-SCD samples.

Figure 6 compares the C K-edge spectra of the bare polished (110) face of SCD (Figure 6a) and the Ni-coated (110) face of SCD (Figure 6b) after annealing and subsequent cooling to room temperature. The spectra display the characteristic features of sp^3^-hybridized carbon, namely, the σ*(sp^3^)-resonance at 289.3 eV and the second gap at 302.3 eV, as well as the π*(sp^2^)- and σ*(sp^2^)-resonances at 285.3 and 291.4 eV of the sp^2^-hybridized carbon. In the spectrum of the Ni-SCD face, the π*(sp^2^)- and σ*(sp^2^)-resonances have lower width and significantly higher intensities (Figure 6b) compared to the spectra of the annealed PCD and SCD, as well as to the Ni-PCD film (Figure 1b). This data indicates that nickel-assisted transformation of the (110) face of SCD produced the graphitic-like coating with a much more ordered structure than that formed on the nickel-coated polycrystalline film. The Raman spectrum of the annealed Ni-SCD sample exhibits a weak D-band and a narrow G-band with the *I*_D_/*I*_G_ ratio of 0.15 (see inset in Figure 6b). This value is lower than that for the annealed Ni-PCD film. Together with the higher intensity of the π*(sp^2^)-resonance in the NEXAFS C K-edge spectrum, this suggests that the defectiveness of the formed sp^2^-hybridized carbon layers decreases as the size of the annealed diamond face increases.

For the annealed Ni-SCD sample, the spectra were measured at angles of 90° and 50° between the photon beam and the sample surface (Figure 6b). This change in the orientation of the diamond crystal relative to the incident radiation from normal to tilted leads to a decrease in the intensity ratio of the π*(sp^2^)-resonance to the σ*(sp^2^)-resonance (*I*_π*_/*I*_σ*_). This is opposite to the dependence for highly oriented pyrolytic graphite [51], and is similar to the dependence for a film of vertically aligned carbon nanotubes [52]. In layered graphite, there is a separation of electron orbitals with respect to symmetry: σ orbitals lie in the basal plane of graphite, while π orbitals are oriented perpendicular to this plane [53]. The difference in the polarization of π- and σ-electrons explains the dependence of NEXAFS spectra of graphitic materials on the angle of incidence of the radiation. Thus, it can be concluded that the orientation of graphitic layers is predominantly vertical relative to the diamond surface.

The intensity ratio *I*_π*_/*I*_σ*_ is 0.67 at an angle of incidence of 90° and decreases to 0.44 with decreasing angle to 50°. These values can be used to quantitatively estimate the disordering of graphitic layers in crystallites. An increase in the width of the angular distribution of graphitic layers results in a weakening of the angular dependence of NEXAFS spectra. Comparing the calculated dependences of the intensity ratio *I*_π*_/*I*_σ*_ [52] with our experimental results, the width of the angular distribution of graphitic layers is no more than 5°, meaning a slight deviation of graphitic layers from the vertical position.

According to [19], graphite layers grow both on Ni particles parallel to their surface and on the etched (110) face of Ni-free diamond at a large angle to the diamond surface. The observed angular dependence of NEXAFS resonances from sp^2^ carbon indicates that the majority of graphite layers is predominantly located on the outermost surface of Ni-free diamond at a large angle, close to a right angle. A similar angular behavior of the NEXAFS spectrum was previously observed for the iron-coated (100) face of a polished SCD after vacuum annealing at 1150 °C [27] and for the (111) face of SCD annealed at 1250 °C [15].

## Conclusion

Polycrystalline diamond films with mixed grain orientations were synthesized by the PE CVD method from hydrogen/acetone/air plasma and coated with a 40 nm thick nickel layer. In situ XPS and NEXAFS data revealed the difference in the chemical state of carbon atoms on the surface of bare and Ni-coated PCD films after annealing in high vacuum at 1100 °C. The temperature used was found to be hardly sufficient to transform the bare surface of the polycrystalline film, while the presence of the nickel catalyst promoted this process, causing the formation of the thin graphitic-like coating. Nickel increases the degree of atomic ordering of the graphite layers formed as a result of the thermal transformation of diamond. The SEM images revealed that nickel particles effectively etch the (110) face, while they mostly stay on the (111) surface, indicating anisotropic diamond etching during heating. Despite these differences, the Raman spectra recorded from the different faces of the annealed microcrystallites were similar, indicating that the carbon coating consisted of graphitic multilayers with a similar structure. The (110) face of SCD was covered with nickel and annealed in high vacuum at 1150 °C. Comparing the NEXAFS C K-edge spectra and Raman spectra of the annealed Ni-SCD and Ni-PCD samples indicates that the amount of defects in the formed graphite layers decreases as the size of the diamond face increases. The best crystallinity of the sp^2^ carbon coating was observed in the case of the SCD substrate. Changing the angle between the synchrotron beam and the flat surface of Ni-SCD revealed a significant increase in the π*(sp^2^) peak intensity at normal incidence. This behavior indicates an anisotropic texture of sp^2^ carbon coating, corresponding to the upright orientation of graphitic layers relative to the (110) face of SCD. Given that the nickel particles coated with sp^2^ layers are primarily embedded within the diamond bulk, this suggests that the topmost surface of the annealed (110) face of diamond is mainly formed through the saturation of bonds on the etched diamond surface by free carbon atoms diffusing from the Ni-diamond interface. This implies that the multilayer graphitic layers formed on the surface of micro-sized crystallites in the annealed Ni-coated PCD are also oriented perpendicular to the crystallite surface. Our results can be useful for controlling the growth of graphitic coatings on dielectric diamond surfaces with a polycrystalline structure and grains of different sizes and crystallographic orientations.

## Experimental

The growth of PCD films on silicon substrates was performed using PE CVD with a hydrogen/acetone/air mixture. The deposition parameters were typical of those previously employed for an “Astex” system (2.45 GHz, 4.5 kW): a pressure of 115 Torr, hydrogen, acetone, and air flow rates of 500, 18, and 0.3 sccm, respectively, and substrate temperature in the range of 940–980 °C [28,37]. The obtained films were about 500 μm thick. Synthetic SCD were produced using high-pressure high-temperature (HPHT) method on a BARS apparatus [54]. The starting materials included a graphite rod (99.99% purity), a Ni_0.7_Fe_0.3_ alloy as a solvent catalyst, and a synthetic diamond (≈0.5 mm) as a seed crystal. The SCD crystals were polished along the (110) plane to obtain the (110)-oriented crystal face. The surface of a PCD film and the (110) face of a diamond crystal were coated with a nickel film using thermal evaporation method (HBA Carl Zeiss Jena setup). The parameters of similar metal depositions are described elsewhere [27]. Nickel was deposited onto the surfaces of the samples for 30 s, resulting in the formation of metallic films with a thickness of about 40 nm.

The thermal transformation of the samples and XPS and NEXAFS experiments were carried out at the RGL-PES end station of the Russian–German dipole beamline (RGBL dipole) of the Berliner Elektronenspeicherring für Synchrotronstrahlung (BESSY II) operated by the Helmholtz-Zentrum Berlin für Materialien und Energie (Berlin, Germany) [55]. Light polarization at the RGBL dipole is linearly horizontal. The samples were fixed in pairs to a molybdenum holder using molybdenum foil strips so that spectra could be recorded from bare and Ni-covered PCD films and bare and Ni-coated SDC with the (110) face directed outwardly. Before the measurements, the samples were annealed in ultrahigh vacuum (10^−9^ mbar) in a preparation chamber of the end station. The annealing was performed at 1100 °C for bare and Ni-coated PCD films and at 1150 °C for bare and Ni-coated SCD samples for 15 min to reconstruct the sample surface. After annealing, the samples were cooled naturally and transferred to an analytical chamber without breaking ultrahigh vacuum conditions.

The NEXAFS spectra of the annealed samples were registered using TEY and AEY modes, which provided complementary information about the chemical state of carbon in the volume and at the surface of the samples. The mean probing depth was estimated to be no more than 10 nm for TEY and 3 nm for AEY. The TEY spectra were recorded by measuring the leakage current with a Keithley ammeter. The experimental data were normalized to the ring current and photon flux measured using a clean gold crystal. In the AEY spectra, emitted Auger electrons were measured using a PHOIBOS 150 analyzer. The polar rotation of the annealed Ni-coated SCD on the manipulator was used to measure the C K-edge spectra at angles of 50° and 90° between a horizontally polarized photon beam and the sample surface. The spectra of bare SCD and PCD samples were measured at angles of 65° and 35°.

The XPS spectra were collected using the PHOIBOS 150 analyzer at photon excitation energies of 330 and 830 eV. Considering the electron inelastic mean free path in solids, the probing depth of the XPS spectra is estimated to be approximately 3 nm for 830 eV and about 2 nm for 330 eV. The energy calibration of the XPS spectra was performed by referring to the Au 4f_7/2_ line at 84.0 eV measured from a clean Au foil. XPS data processing was carried out using CASA XPS software version 2.3.15. Fitting of the core-level spectra was performed using the sum of Gauss–Lorenz and Doniach–Sunjic functions after the subtraction of a Shirley’s background.

The morphology of the clean and Ni-coated PCD film after annealing in high vacuum at 1100 °C was studied using SEM with a JEOL 6700F microscope (accelerating voltage of 5 kV, JEOL Ltd., Tokyo, Japan). EDX spectroscopy analysis was carried out on a Bruker XFlash 6 spectrometer. EBSD analysis of PCD crystalline orientation was performed using a Hitachi S-3400N microscope (accelerating voltage of 20 kV, Hitachi Ltd., Berkshire, UK) equipped with a HKL Advanced EBSD System Nordlys II S. The diffraction patterns were obtained using Flamenco software and analyzed using Tango software. Raman analysis was conducted using a LabRAM HR Evolution spectrometer (Horiba Ltd., Kyoto, Japan). The spectra were excited with a 514 nm laser at a power of 1.9 mW. The laser beam was focused to a diameter of about 1 μm using an LMPlan FL 50×/0.50 Olympus objective. All measurements were carried out at room temperature.

## Supporting Information

File 1 Additional figures and tables.

## Data Availability

Data generated and analyzed during this study is available from the corresponding author upon reasonable request.
