# Supplementary material for: Heat-induced transformation of nickel-coated polycrystalline diamond film studied in situ by XPS and NEXAFS
Source: Beilstein J Nanotechnol. 2025 Jun 12;16:887–98. doi: 10.3762/bjnano.16.67 (PMC12207268; doi:10.3762/bjnano.16.67)
Supplement: File 1 — Additional figures and tables. [file Beilstein_J_Nanotechnol-16-887-s001.pdf]

BEILSTEIN JOURNAL OF NANOTECHNOLOGY

Supporting Information

for

Heat-induced transformation of nickel-coated polycrystalline diamond film studied in situ by XPS and NEXAFS

Olga V. Sedelnikova, Yuliya V. Fedoseeva, Dmitriy V. Gorodetskiy, Yuri N. Palyanov, Elena V. Shlyakhova, Eugene A. Maksimovskiy, Anna A. Makarova, Lyubov G. Bulusheva and Aleksandr V. Okotrub

Beilstein J. Nanotechnol. **2025,** *16,* 887–898. doi:10.3762/bjnano.16.67

Additional figures and tables

License and Terms: This is a supporting information file under the terms of the Creative Commons Attribution License (https://creativecommons.org/licenses/by/4.0). Please note that the reuse, redistribution and reproduction in particular requires that the author(s) and source are credited and that individual graphics may be subject to special legal provisions.
The license is subject to the *Beilstein Journal of Nanotechnology* terms and conditions: (https://www.beilstein-journals.org/bjnano/terms)

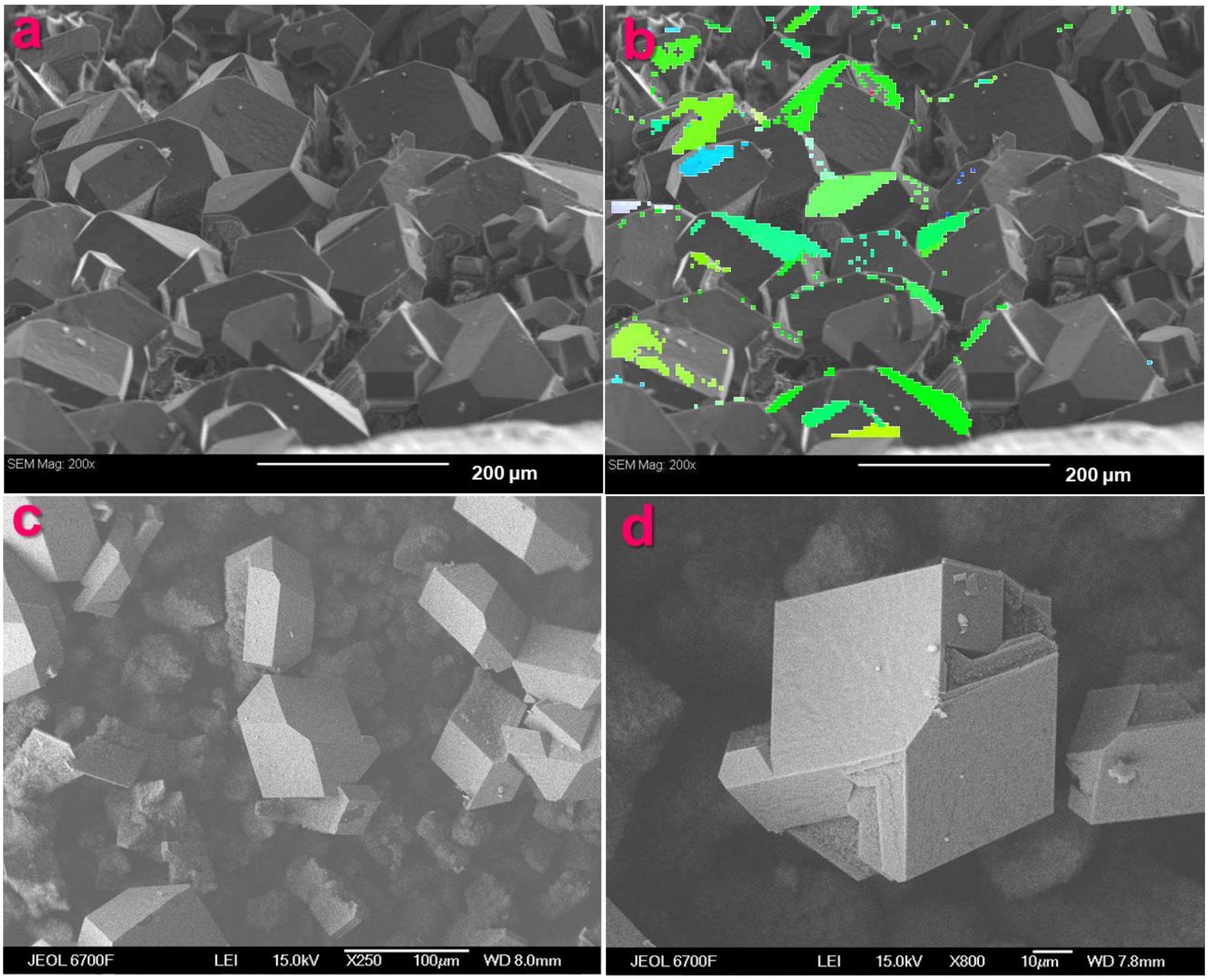

Figure S1: Scanning electron microscopy (SEM) images of the surface of initial polycrystalline diamond (PCD) film (a–c). Panel (b) shows an image with an overlay of the grain orientation map in the colors of the reverse pole figure obtained by electron backscattered diffraction (EBSD) (b), grains with (110) and (111) orientations are shown in green and blue, respectively. SEM image of micro-sized crystallites covered with Ni (d).

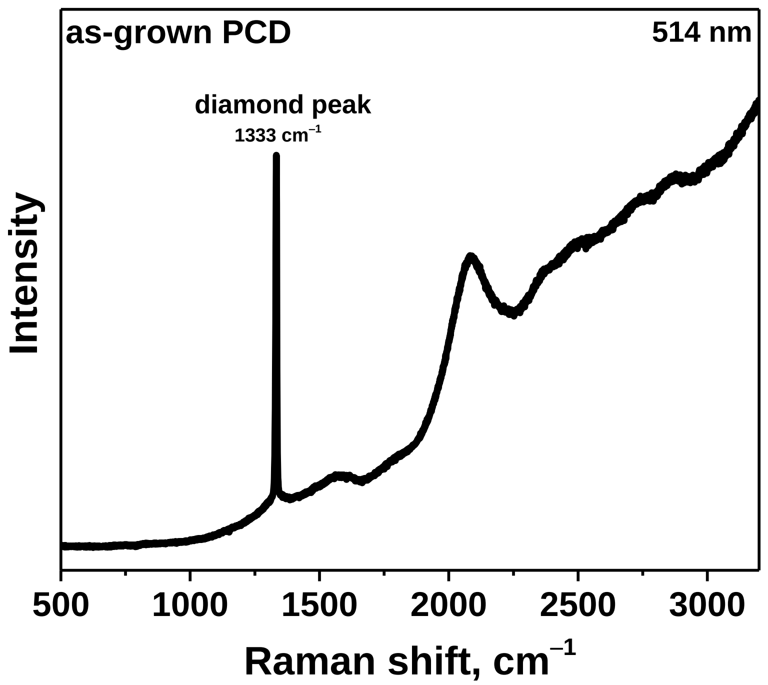

Figure S2: Raman spectrum of as-grown PCD film.

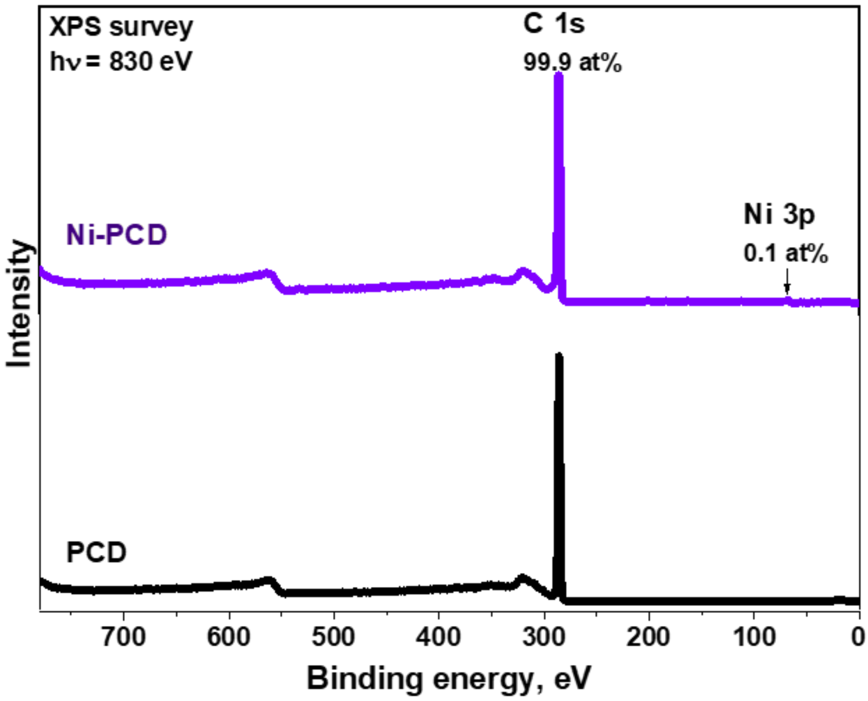

Figure S3: XPS survey spectra of PCD and Ni-PCD films after high-vacuum annealing at 1100 °C for 15 min. The spectra were measured at an excitation photon energy of 830 eV.

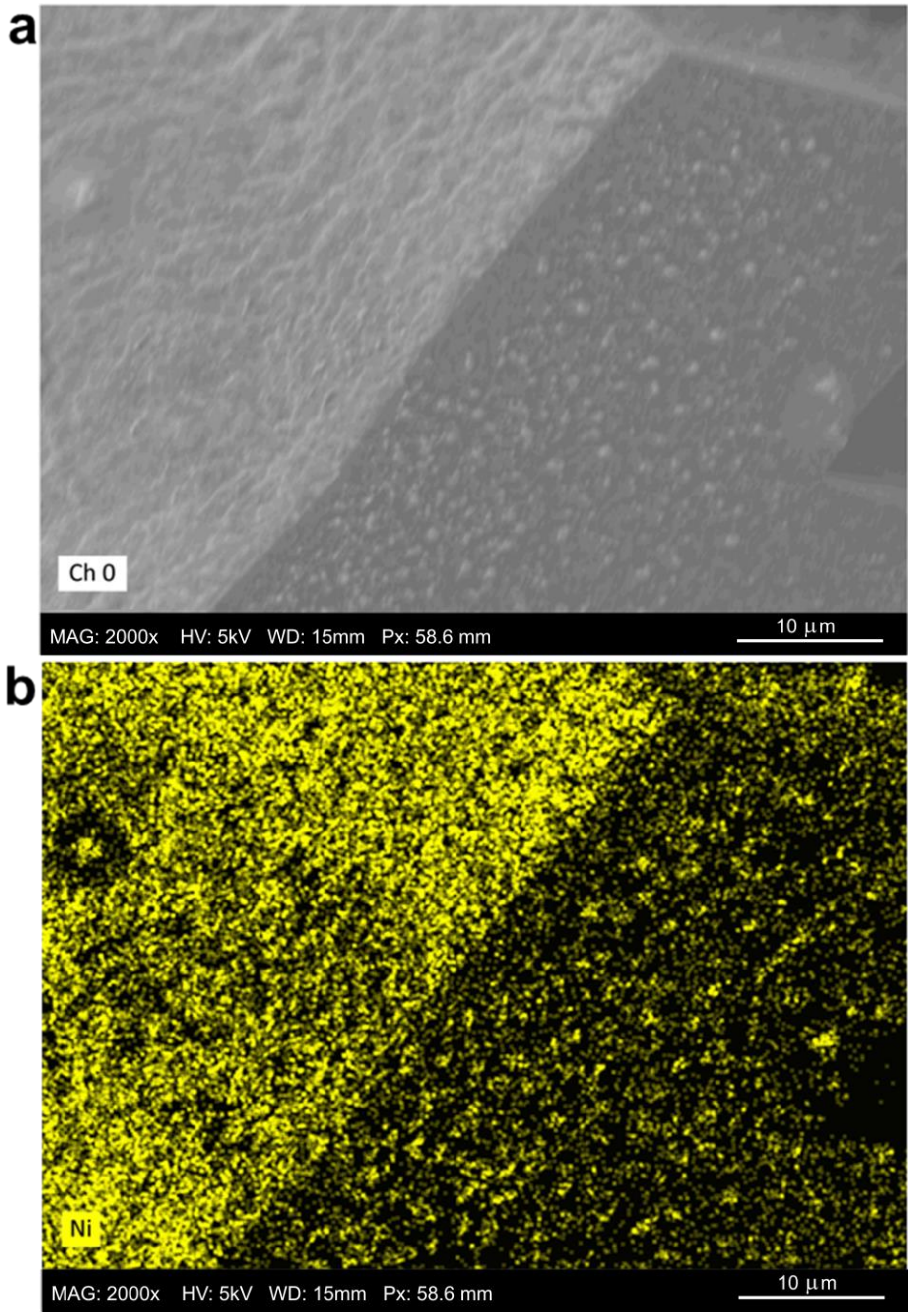

Figure S4: SEM image (a) and EDX map of Ni distribution (b) obtained for annealed Ni-PCD film.

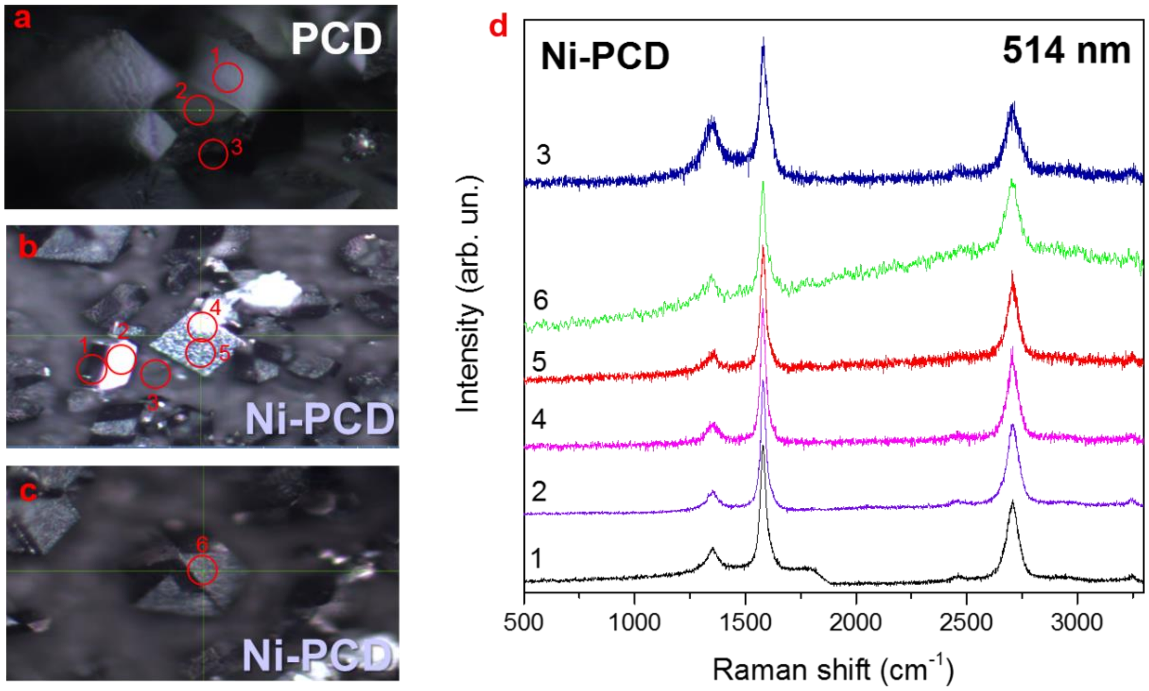

Figure S5: Optical images of PCD (a) and Ni-PCD (b, c) films after high-vacuum annealing at 1100 °C for 15 min. Red ovals indicate the points used for Raman investigation. Raman spectra from regions denoted 1, 2, and 3 in panels (a) and (b) are shown in Figure 5 of the main text; spectra from regions 1–6 of Ni-PCD film (b, c) are shown in panel (d).

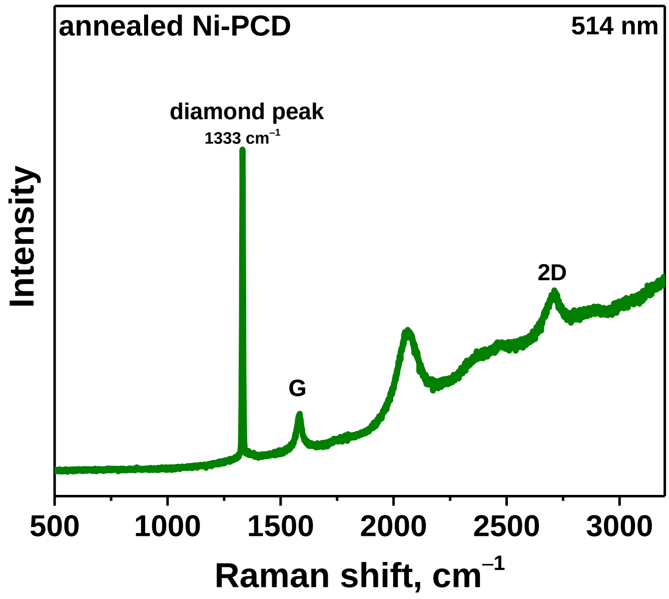

Figure S6: Out-of-focus Raman spectrum of the Ni-PCD film after high-vacuum annealing at 1100 °C for 15 min.

Table S1: Summary on the absorption coefficients (α) of graphite measured at 532 nm using different methods [1]. The probing depth of Raman spectroscopy measurements of graphite-like materials (*d*) can be estimated as follows: $d = 1/2\alpha$.

532 nm	Photoacoustic	Ablation	Ellipsometry
Absorption coefficient (α, 1/μm)	7.7	4.7	5.5
Probing depth (*d*, nm)	64	110	91

References

1. T. Smausz, et al., Determination of UV–visible–NIR absorption coefficient of graphite bulk using direct and indirect methods, Appl. Phys. A (2017) 123:633, DOI 10.1007/s00339-017-1249-y.
